# Chemical constituents, clinical efficacy and molecular mechanisms of the ethanol extract of *Abelmoschus manihot* flowers in treatment of kidney diseases

**DOI:** 10.1002/ptr.6818

**Published:** 2020-07-27

**Authors:** Nan Li, Haitao Tang, Liang Wu, Haitao Ge, Yurong Wang, Honglin Yu, Xiuli Zhang, Jimei Ma, Harvest F. Gu

**Affiliations:** ^1^ Center for Pathophysiology, School of Basic Medicine and Clinical Pharmacy Pharmaceutical University Nanjing Jiangsu Province China; ^2^ Department of Endocrinology Jiangsu Province Hospital of Traditional Chinese Medicine, The Affiliated Hospital of Nanjing University of Chinese Medicine Nanjing, Jiangsu Province China; ^3^ Suzhong Pharmaceutical Research Institute Nanjing, Jiangsu Province China; ^4^ Jiangsu Key Laboratory of Drug Screening China Pharmaceutical University Nanjing, Jiangsu Province China; ^5^ Department of Pharmacology China Pharmaceutical University Nanjing, Jiangsu Province China; ^6^ Department of Nephrology Second People's Hospital, First Affiliated Hospital of Shenzhen University Shenzhen, Guangdong Province China

**Keywords:** *Abelmoschus manihot*, chronic kidney disease, diabetic kidney disease, Huangkui capsule, IgA nephropathy

## Abstract

*Abelmoschus manihot*, also called as “Huangkui” in Chinese, is an annual flowering herb plant in the family of Malvaceae. As a traditional Chinese medicine, the ethanol extract of the flower in *Abelmoschus manihot* is made as Huangkui capsule and has been used for medication of the patients with kidney diseases. Its efficacy in clinical symptoms is mainly improving renal function and reducing proteinuria among the patients with chronic kidney disease, diabetic kidney disease or IgA nephropathy. The possible mechanism of Huangkui capsule treatment in kidney diseases may include reducing inflammation and anti‐oxidative stress, improving immune response, protecting renal tubular epithelial cells, ameliorating podocyte apoptosis, glomerulosclerosis and mesangial proliferation, as well as inhibiting renal fibrosis. In this review, we first described chemical constituents and pharmacokinetic characteristics in ethanol extract of the flower of *Abelmoschus manihot*. We then summarized the clinical and epidemiological relevancies of kidney diseases particularly in the mainland of China and discussed the possible molecular mechanisms of Huangkui capsule in the treatment of kidney diseases. Finally, we prospected further research on cellular and molecular mechanisms and application of this Chinese natural medicine in kidney diseases.

**Abbreviations**
ADA: American Diabetes Association
ADRN: adriamycin‐induced nephropathy
AKD: acute kidney disease
AKI: acute kidney injury
CI: confidence interval
CKD: chronic kidney disease
DKD: diabetic kidney disease
DN: diabetic nephropathy
ESRD: end‐stage of kidney disease
GDM: gestational diabetes mellitus
GFR: glomerular filtration rate
IDF: International Diabetes Federation
IgAN: IgA nephropathy
IR: ischemia/reperfusion
PK‐PD: pharmacokinetics and pharmacodynamics
RAS: renin‐angiotensin system
RCTs: randomized controlled trials
Scr: serum creatinine
T1DM: Type 1 diabetes mellitus
T2DM: Type 2 diabetes mellitus
TCM: traditional Chinese medicine
UACR: urine albumin/creatinine ratio

## 1 INTRODUCTION

As a component and basis of life for producing urine, kidney is an important organ to complete metabolism in the body and to maintain the stability of internal environment. Structurally, the kidney consists of two pea‐shaped organs and perform many crucial functions, including maintaining overall fluid balance, regulating and filtering minerals from blood, filtering waste materials from blood, medications and toxic substances, and creating hormones for production of red blood cells, promotion of bone health and regulation of blood pressures. Kidney diseases, or renal diseases, also known as nephropathy, are damages in kidneys, including primary kidney and secondary to other visceral lesions. There are several types of diseases in kidneys. IgA nephropathy (IgAN) is the most common nephritis in the glomerulus of kidneys (Rodrigues, Haas, & Reich, 2017; Trimarchi et al., 2017; Wyatt & Julian, 2013). Chronic kidney disease (CKD) causes the gradual loss of kidney function over time as shown by glomerular filtration rate (GFR) of less than 60 ml/min per 1.73 m^2^ (Chen 2010; Webster, Nagler, Morton, & Masson, 2017). Diabetic kidney disease (DKD, previously called as diabetic nephropathy, DN) occurred in the patients with diabetes mellitus (Thomas et al., 2015). Acute kidney disease (AKD), also known as acute kidney injury, is marked by the sudden reduction in kidney function within several hours or days (Ferenbach & Bonventre, 2016). Although the etiology, pathology, course and pathogenesis of kidney diseases mentioned above are different, renal fibrosis and gradual loss of nephron are the common pathological links, all these kidney diseases can cause a loss of renal function and may consequently result in kidney failure. The loss of function by 85–90% of normal capacity in kidneys is termed as the end‐stage of kidney disease (ESRD). The patients with ESRD need to be treated with dialysis or a kidney transplant to maintain alive (Kanda et al., 2017; Sumida & Kovesdy, 2017). Kidney diseases threaten human health seriously, also brings heavy burden to the society and family. Thereby, pharmacological intervention for the medicines prescribed to the patients with kidney diseases is of importance, but effective therapy remains limited.

**FIGURE 1:**
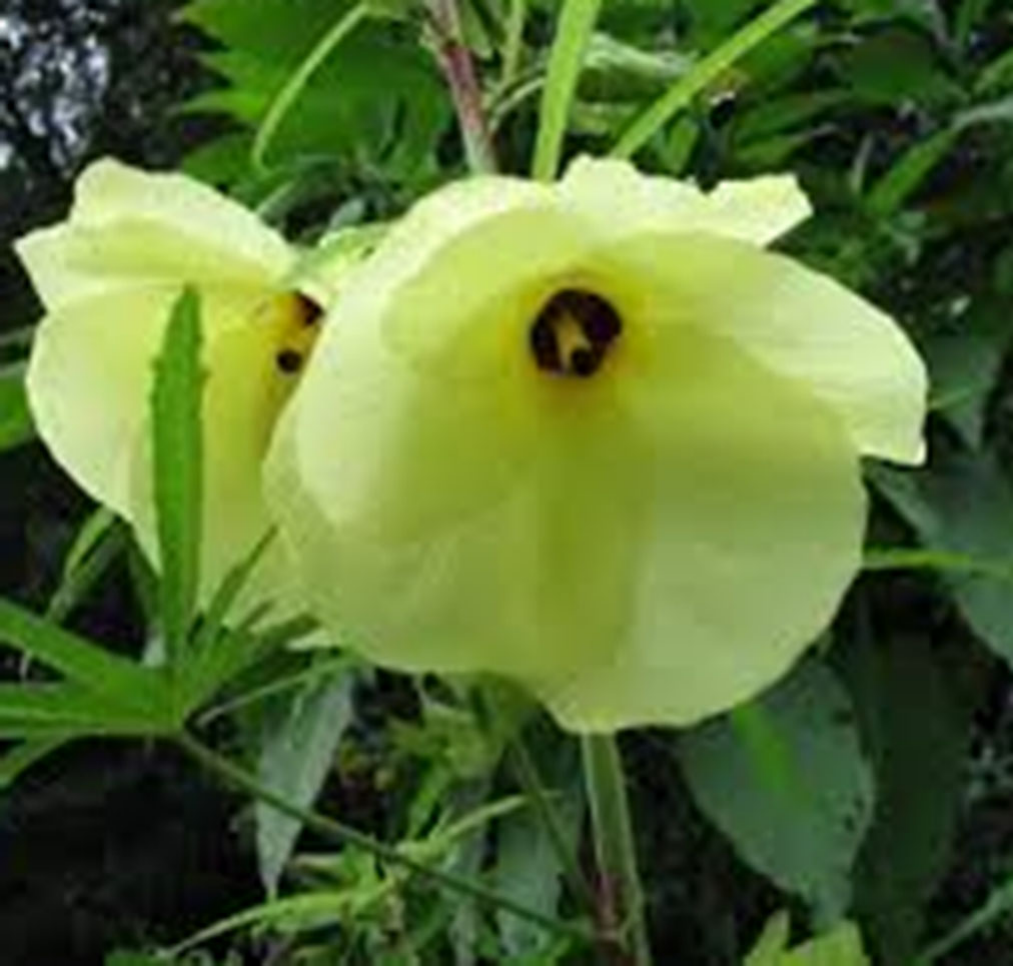
A flower of *Abelmoschus manihot* [Colour figure can be viewed at wileyonlinelibrary.com]

Traditional Chinese medicine (TCM) has a history of over 2,500 years. The herbs associated with TCM are perceived to be a cost‐efficient alternative. As we all known, Ms. Youyou Tu received the Nobel Prize in physiology or medicine in 2015 for her discoveries concerning a novel therapy against Malaria (McPhee & Kenneth, 2016; Zheng, Li, Peng, & Wang, 2020). Actually, she turned to Chinese medical texts mainly from the handbook of prescriptions for emergencies to find a TCM for Malaria, ultimately extracting a compound of Artemisinin, which is a single substance from *Artemisia annua* L. (Zheng et al., 2020). The name of this ancient medical book is Zhou‐Hou‐Bei‐Ji‐Fang in Chinese, and the author is Mr. Hong Ge, an outstanding medical scientist and a senior Taoist doctor in the Eastern Jin Dynasty (317–420 AD), China. From ancient to modern times, some other herbs in China have been found to be effective for treatment of edema, hematuria and symptoms of kidney diseases (Zhong, Menon, Deng, Chen, & He, 2015). One of them is the flowering plant of *Abelmoschus manihot* (Linn.) Medic. (Malvaceae) as seen in Figure 1. It is also known as the sunset muskmallow, sunset hibiscus, or *Hibiscus manihot* in the family of Malvaceae. This plant was formerly considered a species of *Hibiscus*, but is now classified in the genus *Abelmoschus*. Its flowers are yellow in color and look as sunflowers. Therefore, *Abelmoschus manihot* is called as “Huang‐Kui” in Chinese, while “Huang” means yellow and “Kui” is a sunflower. Similar to the discovery story of Artemisinin, the medical application of *Abelmoschus manihot* was also first recorded in the Handbook of Prescriptions for Emergencies by Mr. Hong Ge. Later on, application of *Abelmoschus manihot* had been collected in “Jiayou Materia Medica” [Name of this book in is Jia‐You‐Ben‐Cao in Chinese and prepared by the editor‐in‐chief of Zhang Yuxi and other seven Chinese medical doctors during the Jiayou reign (1,056–1,063) of the Song Dynasty], in “Compendium of Materia Medica” [Ben‐Cao‐Gang‐Mu in Chinese and written by Mr. Shi‐Zheng Li, an excellent Chinese polymath, medical doctor, scientist, pharmacologist, herbalist and acupuncturist in the Ming Dynasty (1,368–1,644) in China] and also in “A Dictionary of Chinese Pharmacy” (edited by Dr. Cun‐Ren Chen and published in 1934) In China, *Abelmoschus manihot* is distributed in the regions of Guangxi, Guangdong, Yunnan, Hunan, Jiangxi, Taiwan and others, and is born in valleys, ditches, roadsides and wild grasses (Pan et al., 2017). The flowers of *Abelmoschus manihot* are generally harvested in summer and autumn. As a TCM, Huangkui capsule is made from the ethanol extract of flowers in *Abelmoschus manihot* and currently used for treatment of the patients with kidney diseases in China.

In this review, we first described the main chemical constituents, pharmacokinetic characteristics and related toxicity studies of *Abelmoschus manihot*. We then summarized the prevalence of kidney diseases particularly in China and discussed the possible mechanisms of Huangkui capsule in the treatment of diseases. Finally, we prospected further development of pathophysiological research and medical application in *Abelmoschus manihot*.

## 2 HUANGKUI CAPSULE AND ITS CHEMICAL CONSTITUENTS, PHARMACOKINETIC CHARACTERISTICS AND TOXICITY

The ethanol extract of the dried flower in *Abelmoschus manihot* is a brown powder, and the taste is slightly sweet and bitter. A previous study with chromatographic and spectroscopic analysis has reported that the flower of *Abelmoschus manihot* contained a total of 13 chemical constituents (Lai, Zhao, & Liang, 2006). Following studies have confirmed that the major pharmacologically bioactive constituents in the flower of *Abelmoschus manihot* are seven flavonoids, including Rutin, Hyperoside, Hibifolin, Isoquercetin, Myricetin, Quercetin and Quercetin‐3‐O‐robinobioside (Guo et al., 2015; Lai, Liang, Zhao, & Wang, 2009). These chemical constituents, their molecular formula and molecular weight are represented in Table 1. Interestingly, Hyperoside and Isoquercitrin are two different chemical constituents in ethanol extract of the flower of *Abelmoschus manihot*. They have the same molecular weight but similar chemical formula because they are isomers.

**TABLE 1:** Chemical constituents of ethanol extract from the flower of *Abelmoschus manihot* in Huangkui capsule

Name	Chemical formula		Molecular weight (amu)
Rutin	C27H30O16	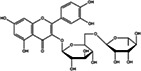	610.52
Hyperoside^a^	C21H20O12	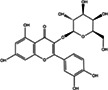	464.38
Isoquercitrin^a^	C21H20O12	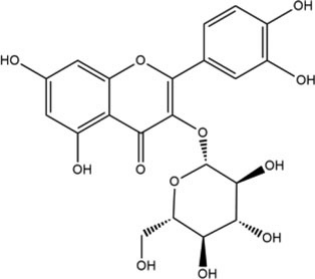	464.38
Gossypetin‐8‐O‐β‐D‐glucuronide, Hibifolin	C21H18O14	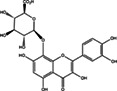	494.36
Myricetin	C15H10O8	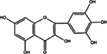	318.24
Quercetin 3‐O‐β‐d‐Glucuronide	C21H18O13	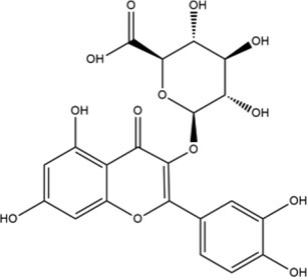	478.36
Quercetin	C15H10O7	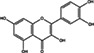	302.24

a They are isomers. Molecular weight is expressed in term of atomic mass units (amu).

Liver and kidney are the main target organs for chemistry component distribution. To test the pharmacokinetic characteristics and toxicity of the flower of *Abelmoschus manihot*, Xue et al. have used orthotopic intestinal perfusion in rats and demonstrated that after oral administration, the flavonoids extracted from the flower of *Abelmoschus manihot* were absorbed by the gastrointestinal tract, while the absorption rates of flavonoids were similar at various drug concentrations. Therefore, the flavonoids is found to be mainly absorbed in the small intestine by passive diffusion, which is a first‐order kinetic process (Xue, Guo, Qian, Duan, & Shu, 2011). Furthermore, Ai G et al. have analyzed the acute toxicity and genetic toxicity of hyperoside from the extract of the flower of *Abelmoschus manihot* and reported that median lethal dose (LD50) of hyperoside was more than 5,000 mg/kg in BALB/c mice without acute toxicity and genetic toxicity (Ai, Liu, Hua, Huang, & Wang, 2013). Yan et al. have further evaluated the protective effects of ethanol extract from the flower of *Abelmoschus manihot* against carbon tetrachloride (CCl4) induced hepatocyte damage in vitro and liver injury in vivo and suggested that extract of *Abelmoschus manihot* may have therapeutic effects for the treatment of hepatic and gall diseases (Yan, Ai, Zhang, Xu, & Huang, 2015). In general speaking, the metabolites of flavonoids in the intestinal tract were delivered to liver through the portal vein for further metabolism, while the flavonoids could be metabolized with hydroxylation, acetylation, methylation, hydrolysis and oxidation loss (Nijveldt et al., 2001). However, our knowledge regarding the interrelationship between pharmacokinetics and pharmacodynamics (PK‐PD) is still limited. It is necessary to establish and improve PK‐PD model for better understanding the relationship between these flavonoids as active ingredients and their pharmacological effects.

As a TCM, the dried flower of *Abelmoschus manihot* had been traditionally used for treatment of the dampness and heat, edema, edema, external carbuncle and inflammation and also for the treatment of skin sores and ulcers, diseases of the urinary system. Until the 20th century, Chinese clinicians had realized that the flower of *Abelmoschus manihot* might have the effect to reduce proteinuria in the patients with kidney diseases. According to the Implementation Standards National Food and Drug Administration National Drug Standard [WS3‐128(Z‐05)‐2003(Z)], Huangkui capsule is made with powder from the ethanol extract of flowers in *Abelmoschus manihot*. Huangkui capsule is composed of 80% ethanol extract, 3% magnesium stearate and 17% calcium hydrogen phosphate. Since 1999, Huangkui capsule as a single medicament of TCM has been used for the treatment of chronic glomerulonephritis, nephritis proteinuria, nephrotic syndrome and the improvement of renal function and inflammatory state of CKD and DKD.

## 3 KIDNEY DISEASES

As described briefly in the introduction, CKD, DKD and IgAN are the main forms of kidney diseases. Other kidney problems include AKI, kidney cysts, kidney stones, and kidney infections are also included. CKD is defined by persistent urine abnormalities, structural abnormalities or impaired excretory renal function suggestive of a loss of functional nephrons. Clinically, CKD refers to all five stages of kidney damage, from very mild damage in Stage 1 to complete kidney failure in Stage 5 (Webster et al., 2017). In the past decades, CKD has been a global public health issue and affects more than 10% population worldwide. Therefore, the burden of CKD is not only restricted to the requirement of renal replacement therapy for ESRD, but also associated with cardiovascular events and mortality.

Diabetes is a group of metabolic diseases characterized by hyperglycemia and has become approaching epidemic proportions globally. Hyperglycemia is caused by defects in insulin secretion or its biological effects, or both. Diabetes is mainly divided into three categories: Type 1 diabetes mellitus (T1DM), which is an autoimmune disease with destruction of pancreatic islet β cells; Type 2 diabetes mellitus (T2DM), which is closely related to insulin resistance and relative insulin deficiency; and gestational diabetes mellitus (GDM), which occurs during pregnancy, and disappears after delivery (American Diabetes Association, 2018; Hunter & Reddy, 2013). In recent years, the global incidence of diabetes has steadily increased. According to the latest report from International Diabetes Federation (IDF), the prevalence of diabetes will be increased from 425 million persons in 2017 to 629 million by 2045 (IDF, 2017). DKD is a microvascular complication and progresses gradually over many years in 30–40% of subjects with T1DM and T2DM (Tuttle et al., 2014). Clinical criteria used to diagnose the subjects with DKD are based upon the presence of albuminuria (two of three specimens of urine albumin/creatinine ratio [UACR] ≥ 30 mg/g collected within a 3‐ to 6‐month period) and/or reduced eGFR (eGFR <60 ml/min/1.73 m^2^) in the absence of signs orsymptoms of other primary causes of kidney damage. Usually macroalbuminuria is defined as UACR higher than 300 mg/g, while microalbuminuria is diagnosed when UACR is between 30 and 300 mg/g. Pathophysiological findings in DKD include glomerular hypertrophy, mesangial matrix expansion, reduced podocytenumber, glomerulosclerosis, tubular atrophy and tubulointerstitial fibrosis. Accumulating evidence has indicated that podocyte loss and epithelial dysfunction play important roles in DKD pathogenesis with further progression associated with inflammation but the exact molecular mechanisms responsible for DKD are not fully known. DKD is a complex disease. Epidemiological studies have demonstrated that there is familial aggregation of DKD in different ethnic groups, indicating that genetic factors contribute to development of the disease. Furthermore, genetic risk factors in DKD interact with the environmental factors (e.g., lifestyle, diet and medication) (Gu, 2019). Clinical observation has implicated that DKD is the main cause of CKD worldwide and the leading cause of ESRD. The presence of CKD is also the single strongest predictor of mortality for subjects with diabetes (Marshall, 2014).

IgAN is also known as Berger's disease and occurs when IgA deposits build up in the kidneys, causing inflammation that damages kidney tissues. IgA is an antibody and this protein is made by the immune system to protect the body from foreign substances such as bacteria or viruses. IgA nephropathy affects the kidneys by attacking the glomeruli. The glomeruli are sets of looping blood vessels in nephrons ‐ the tiny working units of the kidneys that filter wastes and remove extra fluid from the blood. The buildup of IgA deposits inflames and damages the glomeruli, causing the kidneys to leak blood and protein into the urine. The damage may lead to scarring of the nephrons that progresses slowly over many years (Rodrigues et al., 2017; Trimarchi et al., 2017; Wyatt & Julian, 2013). In China, IgAN is the most common CKD. Data from the records in Beijing area, the prevalence of hypertension, intrarenal artery lesions and tubulointerstitial lesions in patients with IgAN at the time of renal biopsy was approximately 40, 55 and 85%, respectively (Xie & Chen, 2008).

## 4 CLINICAL EFFICACY OF HUANGKUI CAPSULE TREATMENT IN KIDNEY DISEASES

Eventually, CKD, DKD and IgAN can lead to ESRD, which is the kidney failure and requires renal replacement therapy such as kidney dialysis or transplantation. Medical management for those kidney diseases before kidney failure is then important to treat the symptoms in the patients with the diseases. Huangkui capsule, as a TCM, has been approved by China's State Food and Drug Administration for the treatment of kidney diseases. Clinical practices by using this medication in kidney diseases have already approved the efficacy because several clinical trials have been done. There are several reports of randomized controlled trials (RCTs) in Chinese hospitals. Five years ago, Zhang L et al. carried out a multicenter study of RCTs. In this study, a total of 417 patients with biopsy‐proven primary glomerular disease from 26 hospitals in China had been participated. The duration of intervention by using Huangkui capsule, 2.5 g, three times per day in these patients was 24 weeks. Results demonstrated that proteinuria in the patients were significantly decreased while mean eGFR did not change, suggesting that Huangkui capsule treatment is a promising therapy for patients with primary kidney disease (CKD Stages 1–2) with moderate proteinuria (Zhang et al., 2014). Two trials with relatively large sizes of the patients have reported. In 2017, Li et al. have conducted another multicenter, prospective, double‐blind, double‐dummy RCT. In this study, approximately 1,600 biopsy‐proven IgAN patients from 100 clinical centers in China were enrolled and followed up for as long as 48 weeks. IgAN patients will be randomized assigned to the *Abelmoschus manihot* group (in the form of a huangkui capsule, 2.5 g, three times per day) and the losartan potassium group (losartan potassium, 100 mg/day). Data indicated that 24‐hr proteinuria from baseline after 48 weeks of Huangkui capsule treatment were decreased, while the estimated glomerular filtration rate (eGFR) from baseline after 48 weeks of treatment increased. Approximately 1,600 biopsy‐proven IgAN patients will be enrolled at 100 centers in China. This study evaluated the efficacy and safety of *Abelmoschus manihot* compared to Losartan potassium in treating patients with IgAN (Li et al., 2017).

DKD initially manifests with microalbuminuria and progresses towards ESRD. Sustained metabolic and haemodynamic perturbations in relation with diabetes may induce subclinical low‐grade renal inflammation and further drive kidney from repair response to damage process, eventually to renal fibrosis. Thereby, the patients with DKD have a diverse range of proteinuria level in urine and kidney function. To evaluate clinical efficacy of Huangkui capsule for treatment in the patients with kidney disease, a total of 5,895 patients (treatment group 3,000 and control group 2,895) from 72 trials have been done in Chinese hospitals or clinical centers. Huangkui capsules, produced by Jiangsu Suzhong Pharmaceutical Group Co., Ltd., have been used for the patients with DKD orally at 2.0–3.0 g three times daily and for 4–24 weeks (Shi et al., 2019). Some of trials are designed by using Huangkui capsule treatment while others with combination of a blocker for renin‐angiotensin system (RAS). Although the trials are ranged differentially and the studies have been conducted in different areas of China, the results have demonstrated that *Abelmoschus manihot* has significant effects to improve proteinuria, while *Abelmoschus manihot* compared to a RAS blocker has more effective. The mean values for 24 hr urinary protein in the patients after treatment are found to be significantly different that is, ‐0.39 g/day (*p* < .001, 95% CI, −0.46 to −0.33), urinary albumin excretion rate (UAER), −19.90 μg/min (*p* < .001, 95% CI, −22.62 to −17.18) and serum creatinine (Scr) −7.35 μmol/L (*p* < .001, 95% CI, −9.95 to −4.76), respectively, but not the rates of adverse drug events (Shi et al., 2019).

Clinical trials have demonstrated that the administration of Huangkui capsule can improve proteinuria and protect kidney function in the patients with DKD, CKD and IgAN, and no obvious side effect is observed. All these studies, however, have been conducted in China, and no trial in other populations has been done. Furthermore, there is no study for the patients with ESRD and it is still unknown whether Huangkui capsule can be benefited in ESRD.

## 5 MOLECULAR MECHANISMS OF HUANGKUI CAPSULE IN TREATMENT OF KIDNEY DISEASES

Although the clinical efficacy of Huangkui capsule treatment in the patients with kidney diseases is significant, the related cellular and molecular mechanisms of this medication are partially but not fully understood. In the past decade, several experimental studies with animal models and/or cell cultures have been reported, and the results are summarized in Table 2. Two previous studies have applied adriamycin‐induced nephropathy (ADRN)‐induced Sprague–Dawley (SD) rats. One group of the rats were treated with Huangkui capsule (2 mg × kg[−1] per day) for 4 weeks. Biochemical parameters in blood and kidneys and the glomerular morphological patterns were examined. Protein expression levels of candidate genes, including alpha‐smooth muscle actin (alpha‐SMA) and collagen Type I, transforming growth factor (TGF)‐beta1, p38MAPK, as well as phosphorylated p38MAPK (p‐p38MAPK) in renal tissues were detected by Western blotting. Results demonstrated that as compared with rats in the untreated model group, the treated rats were found to be improved in urine protein, serum albumin, mesangial cell proliferation, extracellular matrix and collagen deposition. Furthermore, the expression of alpha‐SMA and collagen type I, TGF‐α, TGF‐β1 and p‐p38MAPK in renal tissues was decreased. This study has implicated that Huangkui capsule has the effects in ameliorating renal inflammatory injury in kidneys by reducing TGF‐α, TGF‐β1 expression and intervening p38MAPK signaling pathway (Tu et al., 2013; Zhao et al., 2012). Moreover, a recent study has replicated the experiment with ADRN‐induced SD rats and ADRN‐induced NRK‐52E cells, the normal rat kidney epithelial cell line, and suggested that additional effects of Huangkui capsule treatment could be inhibition of ROS‐ERK1/2‐NLRP3 inflammasomes (Li et al., 2019).

**TABLE 2:** Experimental effects of Huangkui capsule, an extract from *Abelmoschus manihot*, in kidneys and cell‐lines

Kidney disease	Possible mechanism	Experiment	Reference
ADRN	Protect renal tubular cells against ADRN by inhibiting ROS‐ERK1/2‐NLRP3 inflammasomes	ADRN‐induced SD rats NRK‐52E cells	Li et al. (2019)
	Ameliorate renal inflammation by reducing TNF‐α/TGF‐β1 expression and inhibiting p38MAPK signaling	ADRN‐induced rats	Tu et al. (2013) Zhao et al. (2012)
AKI	Attenuate IR‐induced AKI, tubular cell apoptosis, and oxidative stress and inhibit IR‐induced mitochondrial fission by regulating OMA1‐OPA1 axis	IR‐induced AKI mice	Wu et al. (2019)
CKD	Deglycosylation of aglycones such as quercetin, myricetin and gossypetin	CKD rats Intestinal bacteria	Du et al. (2017)
	Prevent tubulointerstitial fibrosis by inhibiting NADPH oxidase/ROS/ERK pathway	CRF rats	Cai et al. (2017)
DKD	Alleviate the early glomerular pathological changes by inhibiting Akt/mTOR/p70S6K signaling	DKD rats	Wu et al. (2018)
	Prevent the kidneys and liver from accumulating pathogenic proteins and dysfunctional mitochondria	DKD mice	Kim et al. (2018)
	Ameliorate renal inflammation by inhibiting the activation of iRhom2/TACE signaling and attenuating ER stress	SD rats HK2 and HRMC cell‐lines	Liu et al. (2017)
	Improve lipid metabolic disorders by activating PPARα/γ and attenuating ER stress	SD rats	Ge, Miao, Sun, and Yu (2016)
	Down‐regulating the activation of p38MAPK and/or Akt pathways as well as the expressions of TGF‐β1 and/or TNF‐α	STZ‐induced rats	Mao et al. (2015)
	Reduce the expression of TGF‐β1 and improve the infiltration and activation of inflammatory cells in glomeruli by intervening p38MAPK signaling	SD rats	Zhao et al. (2012)
	Attenuate Fibronectin and Collagen IV accumulation in renal glomerulus via miR‐21/MMP‐9 axis	db/db mice	Zhang et al. (2016)
	Inhibit glomerular basement membrane damage by inhibiting podocyte heparanase expression	STZ‐induced C57BL6 mice Podocyte	An et al. (2011)
	Improve lipid metabolism via SCAP‐SREBP2‐LDLr signaling pathway in early stage of DKD	db/db mice	Jiang, Yu, Wang, Ge, and Li (2019)

Abbreviations: ADRN, adriamycin‐induced nephropathy; AKI, acute kidney injury; CKD, chronic kidney disease; CRF, chronic renal failure; DKD, diabetic kidney disease; ER, endoplasmic reticulum; HK‐2, human renal tubular epithelial cells; IgAN, IgA nephropathy; IR, ischemia/reperfusion; OMA1, OMA1 zinc metallopeptidase; OPA1, optic atrophy 1; SD, Sprague–Dawley; STZ, streptozotocin.

By using diabetic animal models, a number of Chinese scientists have carried out experimental studies to explore the possible mechanisms of *Abelmoschus manihot* in DKD. Data suggested that the possible mechanisms of *Abelmoschus manihot* in treatment of DKD could be alleviating the early glomerular pathological changes by inhibiting Akt/mTOR/p70S6K signaling, ameliorating renal inflammation by inhibiting the activation of iRhom2/TACE signaling, improving lipid metabolic disorders by activating PPARα/γ, attenuating ER stress and inhibiting the expressions of TGF‐β1 and/or TNF‐α and p38MAPK (Ge et al., 2016; Liu et al., 2017; Mao et al., 2015; Wu et al., 2018). Interestingly, Korean researchers have developed an animal model for DKD by using high‐fat‐diet and streptozotocin (STZ) to evaluate the effects of the supplementation with flower or leaf extracts of *Abelmoschus manihot*. Results indicated that DKD mice showed a significant increase in fasting blood glucose, plasma creatinine, blood urea nitrogen, and urinary albumin levels. Periodic acid^−^Schiff and Sirius red staining of the diabetic kidney presented a significant change in glomerular and tubular structures that was associated with podocyte loss and fibrotic protein accumulation. These changes in these DKD mice were attenuated by treatment with *Abelmoschus manihot*. In addition, hepatic injury, proinflammatory cytokines, and lipid accumulation were decreased after the treatment. This study implicates that *Abelmoschus manihot* can increase the expression of proteins by regulating autophagy and mitochondrial dynamics, which potentially prevented from accumulating pathogenic proteins and dysfunctional mitochondria in not only kidney but also liver of DKD mice (Kim et al., 2018).

In addition to the focus on kidney function, Cai HD et al. have attempted to investigate the effects of *Abelmoschus manihot* in glucose and lipid metabolisms by using the candidant gene approach. They found that five flavonoids from *Abelmoschus manihot* at 5 μmol L^−1^ could accelerate preadipocytes proliferation and regulate the expression of PPARγ, C/EBPα, SREBP‐1, adiponectin, resistin, and visfatin in 3T3‐L1 adipocyte, which implicating that *Abelmoschus manihot* has effects in increasing glucose utilization and improving insulin resistance (Cai et al., 2017). Du LY et al. have investigated intestinal bacteria from normal and CKD rats treated with *Abelmoschus manihot* and suggested that the deglycosylation of aglycones such as quercetin, myricetin and gossypetin could be explained for the effects of *Abelmoschus manihot* in clinical efficacy (Du et al., 2017).

**FIGURE 2:**
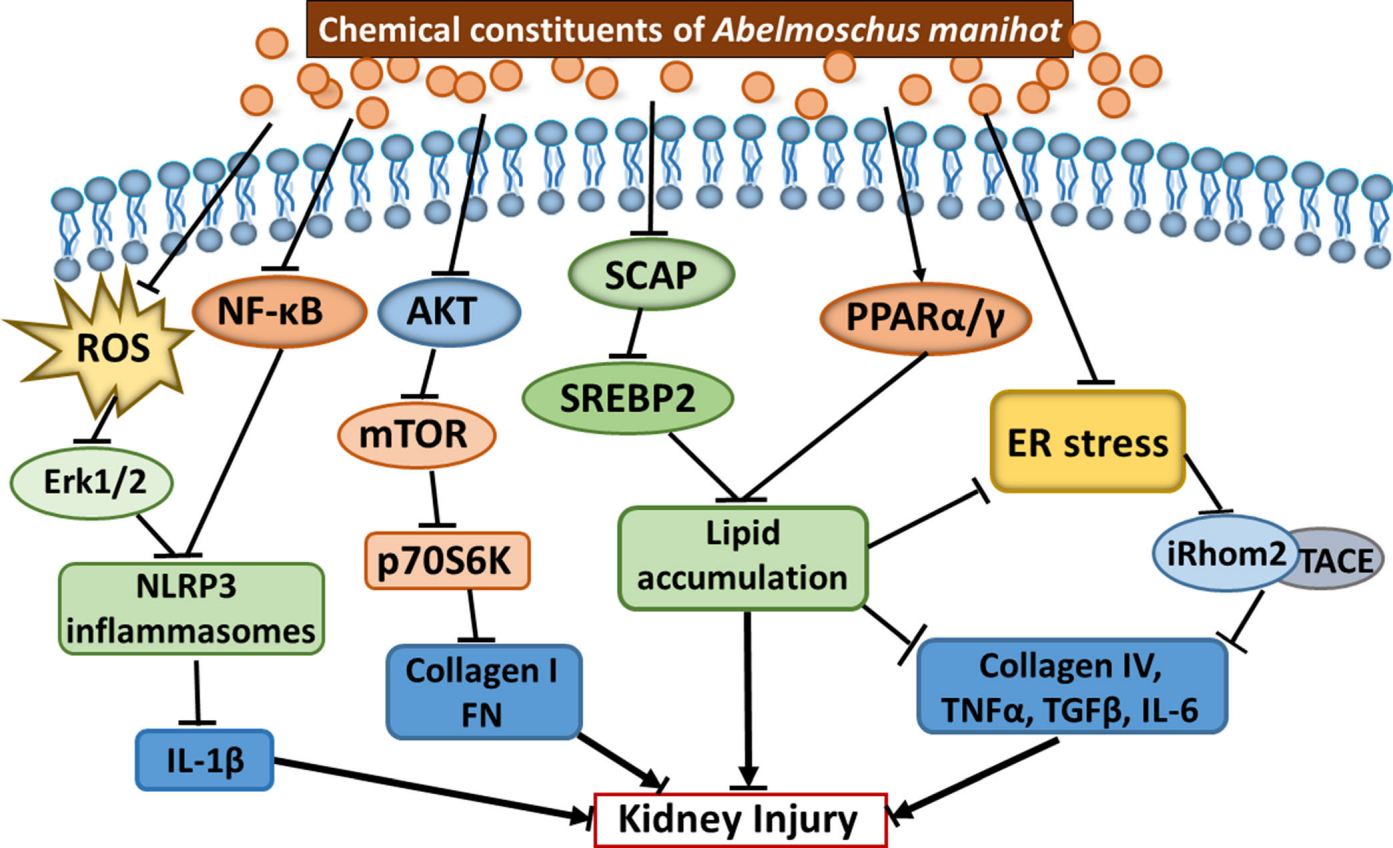
A schematic diagram on possible molecular mechanisms of chemistry constituents in Huangkui capsule for treatment of kidney diseases. This schematic diagram has implicated that the possible mechanisms of Huangkui capsule treatment in kidney diseases may include reducing inflammation, improving immune response, anti‐oxidative stress, protecting renal tubular epithelial cells, inhibiting renal fibrosis, regulating autophagy and mitochondrial dynamics. AKT: also known as PKB, Protein kinase B; ER: Endoplasmic Reticulum; Erk: Extracellular signal‐regulated kinases, also called as MAP kinases; FN: Fibronectin; IL: Interleukin; iRhom2: Rhomboid 5 Homolog 2; mTOR: mammalian Target of Rapamycin; NFkB: Nuclear Factor Kappa B; p70S6K:, Ribosomal Protein S6 Kinase, 70 kDa, Polypeptide 1; SCAP: SREBF Chaperone; SREBP2: Sterol Regulatory Element Binding Transcription Factor 2; PPAR: Peroxisome Proliferator Activated Receptor; TACE: Tumor Necrosis Factor, Alpha, Converting Enzyme; TNF: Tumor Necrosis Factor [Colour figure can be viewed at wileyonlinelibrary.com]

Most of experimental studies have been done by using the extract of *Abelmoschus manihot*, in which seven main constituents are included. It is then valuable to analyze the effects of each chemical compound. Hyperoside is one of active constituents. Wu et al. have recently examined the effects of this constituent in Ischemia/reperfusion (IR)‐induced AKI mice and found that pretreatment of hyperoside could not only attenuate IR‐induced AKI, tubular cell apoptosis, and oxidative stress in the kidneys but also inhibited IR‐induced mitochondrial fission by suppressing OMA1 mediated proteolysis of optic atrophy 1 (OPA1). Furthermore, they found that in human proximal tubular epithelial cells, hyperoside might inhibit CoCl_2_‐induced mitochondrial fission, oxidative stress, and apoptosis by regulating OMA1‐OPA1 axis (Wu et al., 2019). In addition, podocyte injury plays an important role in the occurrence of glomerular insufficiency and proteinuria (Nagata, 2016). An XF et al. have found that hyperoside pre‐treatment inhibited glomerular basement membrane damage in STZ‐induced mice by inhibiting podocyte heparanase expression. The accumulation of lipids, lipotoxicity and lipid metabolism dysregulation are associated with renal injury (An et al. 2017). Jiang et al. have investigated that quercetin improved lipid metabolism via SCAP‐SREBP2‐LDLr signaling pathway in early stage DKD of db/db mice (Jiang et al., 2019). Taking together, the data from experiments with animal models and cell cultures as described above have implicated that the possible mechanisms of Huangkui capsule treatment in kidney diseases may include reducing inflammation, improving immune response, anti‐oxidative stress, protecting renal tubular epithelial cells, inhibiting renal fibrosis, regulating autophagy and mitochondrial dynamics. A schematic diagram implicating the possible molecular mechanisms of Huangkui capsule for treatment of kidney diseases is represented in Figure 2.

## 6 PERSPECTIVE CHALLENGES

Although the figure above has implicated the possible molecular mechanisms of chemistry constituents in Huangkui capsule for treatment of kidney diseases, knowledge and information concerning the pathways underlying the mechanisms still need to be improved. Actually, all previous studies were designed based upon a hypothesis for the certain pathway(s) or candidate gene(s) in relation with kidney diseases. A hypothesis‐driven study, however, is often small‐scale, narrowly focused, and using a limited range of technologies. Therefore, further investigation for better understanding molecular mechanisms of Huangkui capsule for medication of kidney diseases has been taken into our consideration.

As we known that the cases of CKD, DKD and IgAN is increasing, clinical application of Huangkui capsule urgently needs to be broadened and improved. It is then necessary to speed up and improve scientific research to better understand its molecular and cellular mechanisms for the treatment of kidney diseases. However, we are currently facing several challenges. First, the pathogenesis of kidney diseases is very complicated, while CKD and DKD are interlaced, and IgAN is closely related to immunity. The common feature of these kidney diseases is related to inflammation and proteinuria. This may be the breakthrough point for us to further carry out basic scientific research. Furthermore, we need to use the experimental design of hypothesis‐free and genomic research techniques to comprehensively analyze and study the effects of Huangkui Capsule in the treatment of kidney diseases (Evans & Davey Smith, 2015). Second, there are seven major chemical constituents in Huangkui capsule, while Artemisinin is a single substance from *Artemisia annua* L. in Ms. Tu Youyou's discovery (Zheng et al., 2020). TCMs are generally compound prescription drugs. It is still unknown whether they are a single chemical component Huangkui capsule acting alone or in combination with each other to treat kidney disease. Therefore, it is necessary to design experiments for each chemical composition and their different combinations, which inevitably increases the cost and time of the experiment. In order to comprehensively explore the molecular and cellular mechanisms of the treatment of kidney disease by Huangkui capsule, it is necessary to study the interactions and basic regulations of these chemical components in the pathophysiology of kidney diseases. The comprehensive understanding of the mechanism of Huangkui capsule treatment in kidney diseases can expand its clinical application range and enhance its medical value. Moreover, further investigation of Huangkui capsule treatment in kidney diseases may enable us to accumulate knowledge and experience to further understand the basic regulations of interaction for other CTMs and their production of pharmacodynamics. Third, the theory of “gut‐kidney axis” has become a key point to study of interaction of the gastrointestinal tract with kidneys, including immunity inflammation and intestinal bacteria (Chen et al., 2019; Evenepoel, Poesen, & Meijers, 2017). Oral administration of Huangkui capsule into the gastrointestinal tract is the main delivery system of this drug. Our research group has currently designed experiments to investigate the changes of intestinal bacteria and immunity inflammation after oral administration of Huangkui capsule, and data concerning the gut microbiota reprogram and metabolism, the immune and homeostasis resume may provide a novel microbiota‐targeting therapy in medication of kidney diseases.

## CONFLICT OF INTEREST

Haitao Tang, Haitao Ge, Honglin Yu and Jimei Ma are employed by Suzhong Pharmaceutical Group Co., Ltd. All other authors declare no competing interests.
